# A facilitative versus directive approach in training clinical skills? Investigating students’ clinical performance and perceptions

**DOI:** 10.1007/s40037-012-0018-z

**Published:** 2012-06-15

**Authors:** Inneke Berghmans, Nathalie Druine, Filip Dochy, Katrien Struyven

**Affiliations:** 1 Centre for Research on Professional Learning & Development, Corporate Training and Lifelong Learning, University of Leuven, Dekenstraat 2, Box 3773, 3000 Leuven, Belgium; 2 Medical Education Unit, Faculty of Medicine, University of Leuven, Leuven, Belgium; 3 Educational Sciences Department, Vrije Universiteit Brussel (VUB), Brussels, Belgium

**Keywords:** Peer Assisted Learning, Clinical skills training, Clinical learning outcomes, Students’ perceptions

## Abstract

Over the years, many medical school curricula have started implementing diverse student-centred teaching and learning methodologies. Previous studies, however, have indicated that students prefer more traditional and directive methodologies instead, raising questions on which training approach should be advocated. This study contrasts the effects of a student-centred (i.e. facilitative) training approach on students’ clinical skills learning with students’ perceptions. More specifically, a quasi-experimental study was set up in which students experienced either a directive or facilitative training approach. Data were collected by means of an OSCE on the one hand, and a questionnaire on students’ perceptions of the training sessions, and two open-ended questions about students’ likes and dislikes on the other hand. While no general differences were found in terms of clinical knowledge and understanding, and actual clinical performance, an interaction between students’ course-specific prior knowledge and the training approach was found. Especially students with low levels of knowledge benefited more from the facilitative training approach in terms of clinical knowledge, while highly knowledgeable students experienced a negative effect of this training approach. Moreover, students’ perceptions revealed that facilitative-trained students reported more deep-level learning, while the directive training approach turned out to score higher in terms of quality and perceived effects.

## Introduction

The challenge of implementing student-centred learning environments has been placed high on the agenda of many medical school curricula as a primary goal is to stimulate future medical doctors to become self-regulating professionals in the workplace [1, 2]. Medical students need to be prepared to make direct decisions based on a decisive and critical attitude, while taking into account diverse patient- and context-related features. As such, diverse student-centred teaching and learning methodologies, aiming for these competencies, have been introduced into medical education [3]. In this respect, many educators started relying on Peer Assisted Learning (PAL) [4], which is seen as an effective method for stimulating students to take more responsibility for their own learning. More specifically, PAL comprises different strategies that involve the explicit and active support by status equals or matched companions, with the deliberate intent to help others achieve their learning goals (i.e. both knowledge and skills) [5]. Although initially these social-constructivist methodologies were mainly implemented in theoretical courses, over the years PAL has also proven its effectiveness for clinical skills training (practical training), i.e. stimulating better achievement [6], higher self-esteem [7, 8] and better retention of information [9]. However, more process-oriented research is needed to explain its added value in this specific context. As the debate on clinical skills training started focusing more on which training approach would be more beneficial in terms of students’ clinical performance [10], also the training approach adopted by the peer trainer is hypothesized to be important in understanding and stimulating the proclaimed added value of PAL for clinical skills training.

## Theoretical framework

Previous studies in the context of teaching, training and tutoring have offered diverse conceptualizations of approaches to train knowledge and skills [11–15]. Despite terminology differences, a similar underlying distinction is shared between a directive-oriented and a facilitative-oriented approach. A facilitative approach aims to stimulate knowledge construction and deep-level learning by means of questioning and prompting. Instead of giving cut-and-dried answers, the facilitative trainer provides hints and prompts, asks questions and stimulates critical discussion among students. Whereas the facilitative trainer stresses students’ responsibility and adopts a more interactive approach, the directive trainer steers the learning process one-sidedly. He answers students’ questions immediately, supplies them with all the information needed, and demonstrates the course content. As such, students become rather passive participants in the learning process.

To our knowledge, no effect studies have been performed on peers’ adopted training approaches within clinical skills training. The few studies that investigated training approaches within skills lab environments only explored students’ and staff’s satisfaction [10]. In general, medical educators advocate using student-oriented features, such as bouncing back questions and stimulating self-reflection in students. Empirical evidence on students’ actual clinical performance is, however, lacking. Furthermore, Issenberg and McGaghie [16] state that the difference between the acquisition of knowledge and skills should be acknowledged. More specifically, the training of skills asks more explicitly for exposure or observation, practice and assessment. As both training approaches allocate attention to these features, it can be questioned which specific training approach yields the best results.

As instructional effectiveness is stated to be a multidimensional construct [17], it is encouraged to complement findings on actual student learning outcomes with other criteria of effective teaching, such as students’ perceptions of instruction. The importance of students’ perceptions rises because previous studies [18] revealed that students’ perceptions in student-centred settings were less positive as compared with their perceptions of more traditional, lecture-based settings with minimal student responsibility. In other words, the question also arises as to whether the widely advocated power of facilitative training features is agreed upon by students themselves.

## Research questions

1. What is the effect of a facilitative training approach as compared with a directive approach on students’ clinical skills performance?
2. How do students trained by means of either a directive or a facilitative training approach, respectively, perceive the learning environment?

## Methodology

### Setting

The medical faculty of the University of Leuven (Belgium) has introduced a PAL programme into its clinical skills training. Students with skill-specific prior expertise are invited to act as peer trainers for fellow students. Peer trainers are responsible for training small groups of students that are practising diverse technical skills. The educational curriculum of this medical school is characterized by rather traditional teaching methodologies, mainly due to practical reasons that are internationally common within medical school curricula (i.e. staff restrictions, large student population). However, depending on the preceptor, a limited degree of student-centredness is occasionally installed.

### Design

A quasi-experimental study was set up. More specifically, 2nd year Master students (*N* = 363) preparing for their Objective Structured Clinical Examination (OSCE) were randomly assigned to either a directive (*N* = 177) or facilitative trained learning environment (*N* = 186). This group was representative of the general population of medical students (*N*_Male_ = 134, *N*_Female_ = 229), and comprised students who were on average 23 years of age. Students were invited to voluntarily attend eight 1-h training sessions on four different technical skills, i.e. stitching, preparing and giving injections, intravenous catheterization, and bladder catheterization.

A total of 33 peer trainers (*N*_Male_ = 10, *N*_Female_ = 23) at different stages in their training were recruited (*N*_3rdBachelor_ = 17, *N*_1stMaster_ = 4, *N*_2ndMaster_ = 12). Sixteen peer trainers were trained to approach their training sessions in a directive manner, whereas 17 others were trained to approach their sessions in a facilitative manner (Table 1 in Appendix). To rule out possible effects of the stage in training of the peer trainers, a relatively similar number of peer trainers from different years was assigned to each research condition.

The peer trainers received two types of training. Firstly, a medical training refreshed peer trainers’ skill-related knowledge and introduced them to a set of course-related objectives that had to be dealt with during the sessions. Secondly, a 3-h didactical training was organized to prepare peer trainers for their specific role and training approach.

Besides presenting the approach-specific features as such, the training sessions used video examples of good and bad practices, which were discussed in-depth. Also a role play in which peer trainers practised their approach and received feedback and coaching was installed. Furthermore, the first session of every peer trainer was observed by a researcher, after which feedback was given. During the other sessions, the researcher’s presence was guaranteed to provide support when needed. Finally, all peer trainers were given a manual in which possible interventions related to the specific training approach were listed.

Three implementation checks generated proof that the training was well conducted. Firstly, each peer trainer was asked to reflect on their training approach by means of a logbook. Secondly, students were asked to specify which approach they experienced during the sessions. 95.09 % of the students indicated they had experienced the intended approach. Finally, the training sessions were regularly videotaped in order to monitor the approach implemented by the peer trainers.

### Measurements

#### OSCE

The OSCE comprised two parts, i.e. a theoretical test that examined students’ knowledge and understanding (Score_Max_ = 80), and a practical circuit of diverse stations (Score_Max_ = 20). Both parts were administered before and after the eight training sessions.

The theoretical 1-h examination comprised ten questions dealing with patient cases or statements related to the trained skills. The questions were judged by an expert panel as well as by peer trainers to ensure a valid examination. Besides indicating the right answer or stating (dis-) agreement with the given statements (i.e. ‘knows’) [19], students were asked to thoroughly argue their answer in order to also test their understanding (i.e. ‘knows how’) [19]. Each question was assessed by the same corrector on the basis of a correction key that was formulated by an expert panel. In case of ambiguous answers, the expert panel was consulted. As such, a valid correction procedure was developed. Moreover, the study controlled for students’ course-specific prior knowledge and understanding by means of a similar theoretical examination before the start of the sessions. In total, 186 students filled out both the pre- and post-knowledge test (*N*_Dir_ = 93, *N*_Fac_ = 93).

The second part of the OSCE concerned the practical examination. Students had to complete seven diverse stations, including the four skills taught during the training sessions. The experienced assessors were informed about the scoring procedure, but were unaware of which students received which type of training. Following the general OSCE principles [20], assessors were asked to score a list of skill-specific items, as well as to give an overall pass-fail rating. For the purpose of this study, 85 students (*N*_Dir_ = 44, *N*_Fac_ = 41) were randomly selected and assigned to a certain circuit (pretest–posttest). Since not all the students could be assigned to the same circuit of stations, the scores obtained with regards to the skills covered during the quasi-experimental training sessions were averaged and used in the analyses.

#### Students’ perceptions and experiences

To shed light on students’ perceptions and experiences, both quantitative and qualitative data were collected. Firstly, a self-developed questionnaire on students’ perceptions of the peer training sessions was administered, which contained items based on the Course Experience Questionnaire [21] and the Experiences of Teaching and Learning Questionnaire [22], and was supplemented with PAL-specific items. A total of 246 students (*N*_Dir_ = 110, *N*_Fac_ = 136) voluntarily rated 38 items on a five-point Likert scale. An exploratory factor analysis using the maximum likelihood method with orthogonal rotation was conducted. The Kaiser–Meyer–Olkin measure was 0.92, which verified the sampling adequacy for this analysis. Also Bartlett’s test of sphericity, χ^2^(378) = 3008.51, *p* = 0.00, supported this factor analysis. A two-factor solution, explaining 41.15 % of the total variance, resulted from an iterative process in which items that loaded significantly on a factor (≥0.40) were included, and in which cross-loading items were removed until clearly distinctive factors appeared. To corroborate this solution, a new factor analysis on the remaining items was performed, which yielded an identical factor solution (Table 2 in Appendix). Factor 1 (32.15 %) clustered 16 items concerning the degree to which training was perceived to be good and generating diverse added values (‘*Good training and associated effects*’). Factor 2 (9.00 %) contained 12 items relating to the degree to which students felt stimulated towards deep-level and critical learning (*‘Deep*-*level and critical learning’*). Both scales proved to have high internal consistency reliability scores (α_1_ = 0.91, α_2_ = 0.88) and showed a significant positive correlation (*r* = 0.53). For both scales, the scores on all items related to the scale at hand were averaged, generating a scale-specific score on a five-point Likert scale. As was also the case for students’ clinical knowledge, a minimal participation rate of 6 out of 8 sessions was set out as criterion to be included in the subsequent analyses, generating a sample of 242 students (*N*_Dir_ = 108, *N*_Fac_ = 134).

Secondly, all participating students (*N*_Tot_ = 340; *N*_Dir_ = 163, *N*_Fac_ = 177) were asked to indicate what they liked and disliked about the training sessions by means of two open-ended questions: ‘*What did you like about the sessions?*’ and ‘*What did you dislike about the sessions?*’. While the questionnaire was administered at the end of the training programme, the open-ended questions were administered at the end of the course, which was 2 weeks later at the moment of the OSCE.

### Analyses

The quantitative analyses were performed by means of SPSS 16.0. Firstly, to investigate which training approach was more beneficial for students’ acquisition of clinical knowledge and understanding while controlling for students’ course-specific prior knowledge, an analysis of covariance was conducted. Secondly, due to violation of the normality assumption with regard to the practical OSCE performance scores, a Mann–Whitney test was performed. Thirdly, independent *t* tests were preferred to compare students’ perceptions of both learning environments, since the assumption of homogeneity of variance was violated for the scale ‘Deep-level and critical learning’.

The open-ended questions were analyzed using NVivo8 software. With regard to each question, 20 % of the responses were analyzed and coded in order to develop an initial coding scheme. This coding scheme was used to code the rest of the data. As such, an inductive or data-driven coding strategy was used. The coding process was repeated a second time in order to refine the node structure and coding. Finally, a quantitative content analysis was performed, yielding frequency scores.

### Ethics

This study was approved by the ethics committee of the University of Leuven.

## Results

### Students’ learning

**Fig. 1:**
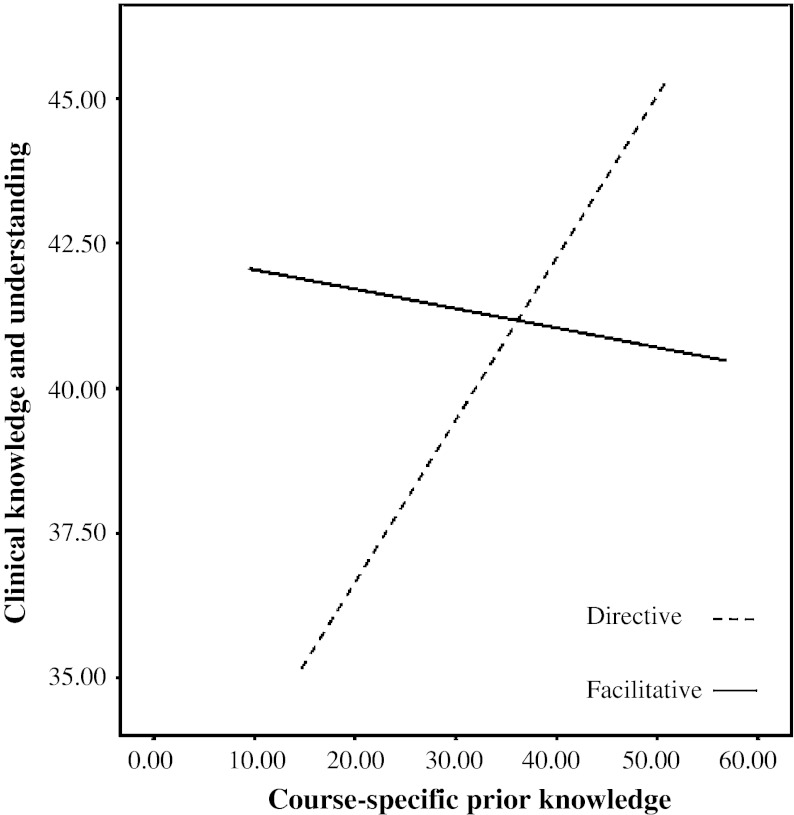
Interaction effect between the training approach and students’ course-specific prior knowledge

The analysis of covariance that investigated the difference in acquired clinical knowledge and understanding between students trained by either a directive (*M* = 40.80, SD = 6.80) or a facilitative peer trainer (*M* = 41.21, SD = 7.20) while controlling for students’ course-specific prior knowledge revealed no significant difference, *F*(1,183) = 0.16, ns. Neither did students’ course-specific prior knowledge differ between the two training conditions, *F*(1,183) = 2.73, ns. However, an interaction effect was found between the training approach and students’ course-specific prior knowledge, *F*(1,182) = 5.33, *p* = 0.02, η^2^ = 0.03 (Fig. 1 in Appendix). Students who started the course with limited prior knowledge turned out to be better off in a facilitative training condition in terms of their acquired knowledge and understanding at the end. Conversely, students who started the sessions with a high level of course-specific prior knowledge experienced a negative influence when being confronted with a facilitative training approach. The latter students scored better in a directive trained learning environment.

Concerning students’ actual clinical performance, no significant difference was found between the facilitative (Mdn = 19.17) and the directive (Mdn = 19) group of students, *U* = 751.50, *z* = −1.33, ns. In other words, directive- and facilitative-trained students scored equally well in terms of their clinical performance.

### Students’ perceptions and experiences

The independent *t* test analyses showed that students trained in a directive manner experienced their training to be of a higher quality (*M* = 4.04, SD = 0.47) than students who were trained by means of a facilitative training approach (*M* = 3.80, SD = 0.56), *t*(1,237) = −3,64, *p* = 0.00, *d* = 0.48. However, facilitative-trained students reported to have been more stimulated towards deep-level learning (*M* = 3.94, SD = 0.47), compared with the students trained in a directive manner (*M* = 3.71, SD = 0.58), *t*(1,203.74) = 3,25, *p* = 0.00, *d* = 0.43. The meaningfulness of these effects is supported by both effect sizes, which are near moderate [23] and practically and clinically significant [24].

The qualitative data provide an in-depth insight into these quantitative findings. Diverse themes of likes and dislikes were reported in both training conditions (Table 3 in Appendix).

### Likes

In their theory-driven curriculum, both groups of students usually appreciated the opportunity to put their knowledge into practice. Furthermore, although directive-trained students commented relatively more on their peer trainers, agreement between the two groups existed in terms of reported likes, i.e. describing their peer trainers as being ‘*friendly*’, ‘*helpful*’, and ‘*motivated*’. In addition, directive-trained students added that their peer trainers were *easily accessible*, were *‘hands*-*on experts at their level’,* and were ‘*well prepared*’. Differences were mainly noticed in terms of reported added values. The directive group liked the clear explanations and clarifications given, and the possibility to ask questions. On the other hand, facilitative-trained students mainly liked that they were stimulated to approach their learning with a deeper and more critical attitude. Related to this, also the fact that they were stimulated to think for themselves was appreciated. Finally, also the questioning strategy of trainers as such scored relatively highly in terms of likes among these facilitative-trained students. Interestingly, whereas the latter students barely mentioned the good atmosphere, students trained in a directive manner mentioned this as the fourth most-liked feature of the training sessions.

### Dislikes

The negative remarks of directive-trained students mainly concerned their peer trainers. In particular, they disliked differences between trainers in general (i.e. personality, motivation) and questioned trainers’ expertise level. This was possibly due to the contradictions experienced during the sessions, which caused some students to experience confusion and no structure while others started to doubt the quality of information and clarifications provided by peer trainers. Finally, directive-trained students also disliked more practical-organizational aspects, such as the syllabus, the infrastructure, and the timing of the sessions.

In contrast, students trained by means of a facilitative approach mentioned more in-depth critical remarks. Their negativity became especially clear with respect to the questioning and prompting strategies of their peer trainers. These strategies resulted in a loss of overview and decreased the actual practice time as extended discussions resulted from these strategies. Moreover, students expected to receive step-by-step instructions during the sessions and, as such, did not prepare beforehand. This made it harder for them to deal with trainers’ questions. Consequently, discussion time increased while the actual practice time was cut down. Secondly, this group also disliked trainers’ explanations and their lack of uniform explanations in particular. As a result of these substantial dislikes, facilitative-trained students often mentioned being annoyed or irritated. Finally, while the majority of negative remarks among directive-trained students dealt with the expertise of peer trainers, only 5.25 % of facilitative-trained students’ dislikes referred to this feature. It seems that directive-trained students made higher demands upon peer trainers’ expertise as compared with facilitative-trained students who mainly disliked the educational strategies adopted by their peer trainers.

## Discussion

Throughout the years, it has been argued that student-centred teaching and learning environments are the best way to prepare students for the medical profession [1]. The current study combined two perspectives to shed light on this notion within the specific context of clinical skills training, i.e. ‘hard’ outcome measures in terms of students’ clinical learning performance, and ‘soft’ learning outcomes in terms of students’ perceptions.

Investigating the effects on students’ actual clinical performance, this study must conclude that the training approach does not generate a differential effect. An explanation for this result may lie in the highly standardized nature of clinical skills. Not only their educators but also their future patients expect students to execute the technical procedures exactly as prescribed by the standardized rules. The high median scores of both groups of students, which are near the maximum score, support this: students tend to know the procedures by heart. The training approach as such does not add much value to that. However, with regards to students’ clinical knowledge and understanding, the widely advocated facilitative training approach turned out to be more beneficial for students who attended the course with limited course-specific prior knowledge. An explanation may concern the student-oriented perspective adopted by the facilitative trainer. As this trainer adapts his questioning and prompting to the individual learner, individualized structure and guidance is guaranteed. Tailoring clinical skills training more to the needs of students is stated to be an important assignment for educational curricula [10]. Although other studies claim that direct instructional guidance is needed for novice or low-ability learners [25], this study proposes that the facilitative approach might also be considered to be a step-by-step guidance, i.e. question-by-question, within the student’s zone of proximal development. In this respect, facilitative training might work better for students with limited course-specific prior knowledge whose schemata fail to give structure. In contrast, prior knowledge schemata of more knowledgeable students are stated to offer sufficient internal guidance [25], which might have minimized the added value of facilitative training for these students. Moreover, at a certain level facilitative training turned out to be even detrimental for highly knowledgeable students. This indicates that when students have reached a certain degree of ‘expertise’, facilitative questioning and prompting strategies no longer provide extra benefit. An explanation could be that these students only ask questions when they really do not have a clue. As such, these students might have perceived strategies such as prompting or questioning to be unnecessary. Consequently, this might have negatively influenced their motivation in this type of training context. Furthermore, their questions are hypothesized to be cognitively demanding. At this level, students might prefer their tutors to give them specific answers or elaborate on the proposed issues in order for them to be stimulated to engage in further reasoning and discussion.

The results on which training approach is more beneficial from the perspective of students support the widely advocated added value of the facilitative training approach. Facilitative-trained students reported to be triggered to think more deeply and critically about the skills. As medical education is considered to be an ‘applied hard discipline’ in which students are expected to apply theoretically acquired ideas to the professional context [26], this finding is especially crucial in this specific context. However, in making important and immediate decisions, it is vital to understand the rationale behind this rather linear sequence of principles or actions. Hence, adopting a flexible and critical thinking attitude becomes crucial. In contrast, students were less positive about the facilitative training approach as such, compared with their directively trained peers. The greatest dissatisfaction resulted from peer trainers’ intensive questioning and prompting, and from their lack of explanations and information. This could be explained by the fact that, in general, medical students are more used to information transmission and directive teaching, compared with constructivist or facilitative teaching [26, 27]. As was also the case for the current research sample, the confrontation with a student-centred training approach generated initial adjustment problems and feelings of frustration. The incompatibility with their preferences for learning and teaching could have lowered students’ appraisal of trainers [28]. In contrast, directive-trained students tended to make higher demands on their peer trainers in terms of knowledge and expertise as compared with facilitative-trained students. Similarly, the directive group might have been comparing peer trainers’ knowledge with the level of knowledge and expertise to which they were used, i.e. of their highly knowledgeable professors.

## Limitations

Practical constraints made it impossible to administer an OSCE to all students in a pretest–posttest phase. Hence, a random group of students was selected to participate. In addition, although a satisfactory number of students voluntarily completed the questionnaire on students’ perceptions (68 %), the group of non-respondents concerned students who were absent during the final session. Reasons for dropout, however, were related to personal and leisure activities. Nevertheless, we did compensate for losing dropouts’ opinions by administering two open-ended questions about students’ likes and dislikes at the time of the examination, generating a 95 % response rate. Finally, the authors wish to encourage researchers to execute similar research in other medical educational settings and curricula. Since this study dealt with peer trainers, the influence of the peer status might have interfered. Accepting a questioning and prompting approach from a clinical teacher is one thing, but to accept this from a peer who is by definition there to help you, is something else. As such, the debate on which clinical training approach is to be preferred can benefit strongly from more empirical evidence in settings using both peers and non-peers as trainers.

## Conclusion

This study concludes that in the context of clinical skills training no single best training approach exists. Trainers are challenged to differentiate and adapt their training approach to the specific student group in terms of course-specific prior knowledge. Moreover, the shift towards student-centred education and the related role change of educators from a director to a facilitator of learning [1] appears to be not well received by medical students. Since students’ preferences are an important matter of concern, these findings raise the question whether medical education needs to respond to students’ clear training or teaching preferences, even if this approach does not make students adopt more deep-level learning strategies. Students’ preferences for learning and teaching should, however, not be viewed as stable as they are open to change [28]. Nevertheless, such a change remains unlikely if educators keep on meeting students’ expectations and preferences. If the development of deep-level learning competencies is in fact high on the agenda of medical school curricula, they have to face the challenge of finding a balance between meeting students’ preferences and stimulating more student-centeredness in learning.

## Essentials

- Directive- and facilitative-trained students score equally well in terms of both understanding and performing clinical skills.
- Differentiating on the basis of students’ course-specific prior knowledge is, however, crucial when stimulating a profound understanding of clinical skills.
- In terms of adopting more deep-level and critical learning attitudes, the facilitative training approach is experienced by students to have an added value.
- Medical students perceive directive training (i.e. cut-and-dried instructions and demonstrations), however, as more positive.
- An added value exists in combining a ‘hard’ outcome perspective (i.e. students’ actual learning outcomes) with a ‘soft’ outcome perspective (i.e. students’ perceptions and experiences) when evaluating a clinical learning environment.

## Appendices

See Tables 1, 2, 3 and Fig. 1.

**Table 1:** Two conceptualised training approaches

	Directive approach	Facilitative approach
	The peer trainer	
Starting the session	Demonstrates the course content and objectives	Asks a student to demonstrate the course content and objectives
Spontaneous trainer actions, including sharing trainer’s own experiences	Gives (extra) information concerning the course content and/or shares his own experiences to deepen understanding	Questions and challenges students concerning the course content and/or his own experiences to deepen understanding
Reaction to students’ questions	Answers questions and insecurities with clear-cut answers	Answers questions and insecurities with questions, hints and/or prompts
Feedback	Gives specific and immediate feedback	Asks students to provide feedback themselves and/or their peers, supported by asking guiding questions
Closing the session	Summarizes the course content, points out important aspects, misconceptions, and/or the mistakes made by students at the end of the session	Asks students to summarise the course content at the end of the session by pointing out one or more things they have learned during the session

**Table 2:** Items and factor loadings of the two factor solution extracted from the exploratory factor analysis on the questionnaire concerning students’ perceptions of the training sessions

Items	
Factor 1 (α = 0.91)	Factor 2 (α = 0.88)
I experienced the support from the peer trainers as being of a high-quality (0.80)	The training sessions have prompted me to think for myself about the skills and the reasons behind these skills (0.72)
I am satisfied with the support received from the peer trainers during the training sessions (0.78)	I think that I learned a lot about the rationale behind certain skills and techniques (0.66)
My peer trainers were extremely good in explaining things to us (0.77)	The training sessions have stimulated me to think more critically about my own actions as a doctor when executing skills (0.66)
I would rather have had a different type of training (−0.66)	The training of the peer trainers has stimulated me to think about the rationale behind certain skills and techniques (0.64)
I think that the peer tutors prepared me well for the OSCE (0.65)	The peer trainers stimulated me to rethink my understanding of some aspects of the subject (0.60)
I enjoyed the training of the peer trainers (0.61)	The training sessions stimulated me to ask questions and to express concerns about the course (0.59)
After finishing these training sessions, I feel well prepared for the OSCE (0.61)	The training sessions have stimulated me to adopt a critical attitude as regards the skills to be learned (0.59)
The peer trainers did their best to make the course content as interesting as possible (0.60)	The training sessions have stimulated my interest for these skills (0.59)
The peer trainers gave useful feedback on how I was progressing (0.59)	The training sessions have stimulated me to think about my learning process, and more especially about how well I was learning and what still needed to improve (0.54)
I learned a lot from the training sessions (0.59)	Throughout the training sessions I learned the relevance of the course content to be learned (0.48)
The training sessions added to a better preparation for the OSCE (0.58)	The training sessions really tried to get the best out of all students (0.48)
I think that I learned a lot about the skills and related techniques (0.58)	The training sessions were enjoyable (0.43)
My self-confidence concerning the skills to be acquired has grown due to the training sessions (0.44)	
The peer trainers’ training approach allowed me to ask questions more easily (0.44)	
The training sessions have given me a good overview of the skills (0.44)	
The peer trainers spent a lot of time giving feedback on our progress (0.44)

**Table 3:** Students’ likes and dislikes about the training sessions (reference coding)

	Directive trained students	%	Facilitative trained students	%
Likes	Opportunity to practice	24.15	Opportunity to practice	18.58
	Peer trainers: friendly, helpful, motivated, easily accessible, ‘hands-on’ experts, and well prepared	19.28	Being stimulated to adopt a critical attitude towards the course and deep-level learning	10.62
	Clear explanations and clarifications	12.34	Being stimulated to think further for myself	9.07
	Good atmosphere during the training sessions	7.71	Peer trainers: friendly, helpful, motivated	8.85
	Possibility to ask questions	6.17	Clear explanations and extra info of which students were unaware	6.86
			Questioning strategies	5.53
Dislikes	Peer trainers: expertise and knowledge, and inter-individual differences	11.02	Questioning and prompting of peer trainers	21.22
	Contradictions among peer trainers, within syllabus, and/or between syllabus and peer trainers.	7.99	Lack of (uniform) explanations and information	8.82
	Quality of syllabus	8.26	Quality of syllabus	6.09
	Infrastructure	7.16	Frustration/irritation	5.46
	Timing of sessions	6.06	Limited practice time	5.25
	Quality of information and clarifications	5.79	Peer trainers: expertise and knowledge, and inter-individual differences	5.25
